# The Responsibility of Health on the High Seas

**Published:** 1908-04

**Authors:** 


### The Responsibility of Health on the High Seas

THE question of responsibility of the health and well-being of passengers and crews while traveling on the high seas is a most important one, and yet that responsibility seems to be divided. The captain and steamer surgeon seem to possess supreme authority, and yet in many cases epidemics and unsanitary conditions are overlooked and loss of life, and great inconvenience are inflicted upon crew and passengers alike. It might be well to suggest a change in the order of things, and make the steamer liable to the health regulations of the port which clears her or receives her. Crimes committed upon the high seas are punishable according to the laws of the state or country toward which a vessel is bound. By act of Congress, due to the energy of Major Farquhar, then a representative from Buffalo in the lower House of Congress, the Great Lakes were declared to come under the ruling of the high seas, and subject to all laws and regulations pertaining thereto.

The Marine Hospital service, with its chain of hospitals along the Great Lakes, has performed important service in looking after the health and comfort of sick and disabled sailors, but it should also have supervision over the sanitation of the vessels plying the Great Lakes as well. Then with the active coöperation of the health physician of her clearing or receiving port, the sanitary conditions would be thoroughly investigated and abuses corrected.

During the summer of 1907 the steamer "Northwest" of the Northern Navigation Company, plying between Buffalo and Duluth, was in such an unsanitary condition that an outbreak of typhoid fever followed. The matter was taken up by the Council of the Buffalo Academy of Medicine and some correspondence passed between Dr. Walter Wyman, Surgeon General of the Marine Hospital Service, Congressman Alexander of Buffalo, and the Council of the Academy. An investigation followed which, however, is not yet completed and results are not ready to be published.

It seems as if the officers of this company would take every precaution, which they undoubtedly have, to prevent a like occurrence, because of the great popularity of which these vessels enjoy by the traveling public—their sumptuous arrangements and the superiority of those vessels to any others on the Great Lakes. A trip on the "Northland" or "Northwest" is as delightful as an ocean voyage—with all the comforts, luxuries and appurtenances of the ocean liners—and the passengers must have a sense of safety in order to enjoy to the fullest the rare treat offered by the greatest of inland water trips.

